# The Diseases of the Ear, Their Diagnosis and Treatment

**Published:** 1864-06

**Authors:** 


### REVIEW.

*The Diseases of the Ear, their Diagnosis and Treatment. By* Dr. Anton Von Troltsch. *From the German, New York:* Wm. Wood & Co.,—1864.

This book has been translated from the German by D. B. St. John Roosa, M. D., of New York, and by so doing he has conferred a great favor upon the medical profession. There are few works devoted especially to diseases of the ear, and perhaps it may be truthfully said, that there is not in the whole range of medical and surgical knowledge, any one subject of equal importance so poorly understood by medical practitioners throughout the country, as the diseases of the ear and the means by which they may be relieved or removed.

This book is believed to be founded upon pathological investigation and ample experience, and to contain some views not found in any other work upon diseases of the ear.

The method of illuminating the external ear is the best in use, and certainly deserves attention; it is fully illustrated by wood cuts. In the practice of aural surgery there is unquestionably much to be learned, and the appearance of this book is an indication that attention is being attracted to the subject, and though all the opinions expressed may not prove correct, still the views are founded upon careful observation and appear to be well sustained. We most heartily recommend the work to our readers, and believe that it will be found worthy their attention and confidence.

Published by Wood & Co., New York. For sale in Buffalo by Breed, Butler & Co.

